# Supplementary material for: The dynamics of entrepreneurial agglomeration formation: Social selection and simulation
Source: PLoS One. 2023 Sep 28;18(9):e0291615. doi: 10.1371/journal.pone.0291615 (PMC10538723; doi:10.1371/journal.pone.0291615)
Supplement: Attachment — Submitted filename: Response to REVIEWERS.docx [file pone.0291615.s003.docx]

There are two reviewers who have checked our paper. Reviewer 1 provides some suggestions on our modification, and the Review Comments to the Author from reviewer 2 is “nothing”. Therefore, our responses to the comments are mainly focused on reviewer 1.

**Reviewer #1: This paper is related to an interesting topic, namely The Dynamics of Entrepreneurial Agglomeration Formation: Social Selection and Simulation for PLOS ONE. However, the manuscript needs to be further developed in order to meet the expected academic requirements.**

Response: Dear reviewer, we highly appreciate your efforts for reviewing our paper and giving valuable suggestions. It really helped us to improve the quality of paper.

**1) Separate Conclusion from Suggestion
Add limitations and direction for future research in new section.**

**Response:** Thank you for the comments. We have separated **Conclusion from Suggestion**, and added **Limitations and Direction for Future Research** in new section (under Section 5, page 25-28, marked in red)

*5. Conclusion and Suggestion*

*5.1 Conclusion*

*The entrepreneurial agglomeration, as an effective means to sustain regional economic development, has been widely emphasized globally, especially in emerging countries such as China. However, numerous cases show that the fostering and consolidating entrepreneurial agglomeration is not an easy task. For lots of regions with similar social and economic circumstances, the ultimate results for the entrepreneurial agglomeration vary dramatically though the local governments have paid great endeavors in forging them. Then we are facing the problems of “what are the factors that determine the entrepreneurial agglomeration”, “why entrepreneurship mostly prospers in some regions while not in other regions?” and “why the region with similar situations cannot replicate the entrepreneurial agglomeration as others?”*

*In order to explore the determinants of entrepreneurial agglomeration formation, traditional literatures have mostly sought evidences from the individuals or the regional conditions. However, as our observation, the individuals cannot clearly obtain and understand all necessary information and resources for entrepreneurship in the given region. That is to say, traditional information for the new entrants is generally ambiguous. Along with this judgement, the individuals will seek for much easier criterion to make their career choices. Entrepreneurial agglomeration is reflected as the aggregated entrepreneurship. For any region, the new entrant is fully exposed to the environment where he is with some probability to meet the entrepreneurs, and the bandwagon effect will drive him much easily affected by his proximity to take similar activity. Under such argument, we provide a theoretical framework describing the dynamics of entrepreneurial agglomeration formation. Unlike the traditional research that assume the individuals are informed all the necessary information in the given region, we introduce the hypothesis that the new entrants will make their career choices (entrepreneurship or employment) based on existing entrepreneurial ratio. According to our model, after repeated choices, this trajectory conforms to a nonlinear Polya process, i.e., the whole process will emerge the self-reinforcing and path-dependency characters. Then after numerical calculation and simulation, we confirmed that the process under our assumption will converge to some fixed points, which can exhibit the formation of entrepreneurial agglomeration and explain why different regions will vary in their levels of entrepreneurial agglomeration.*

*Our main findings are concentrated on the discovery of the dynamic process of and the determinants for the formation of entrepreneurial agglomeration. Under our assumption, our model can explain the formation of entrepreneurial agglomeration is a nonlinear Polya process, and the repeated entrants’ career choices will lead to the convergence of entrepreneurship in any given region. As to the determinants, our analysis and simulation have proved at least four categories can be included: the agent’s risk compensation value, the initial scale of agents, the number of role models (especially in the early stage), and the initial entrepreneurial ratio. Accordingly, they can basically answer three questions in section 1.*

*5.2 Implications*

*Our findings have some theoretical implications: it is proved that entrepreneurship has “memory” and the entrepreneurial history could have influence on the future, which can be expressed as another kind of bandwagon effect emerging from entrepreneurial activities; it is concluded theoretically that 4 determinants from agents and regions, i.e., the agent’s risk compensation value, the initial scale of agents, the number of role models (especially in the early stage), and the initial entrepreneurial ratio, can contribute to the formation of entrepreneurial agglomeration, thus focusing on improving individual’s risk-taking abilities, increasing the initial population scale and entrepreneurial ratio in the region, and introducing more entrepreneur role models in the early age can improve the formation of entrepreneurial agglomeration.*

*Also, our findings have some practical implications. Considering the strong effects from “memory” and “history” in entrepreneurship, our policy should be more concentrated on the determinants from “early” age, such as attracting more role models and introducing more entrepreneurial activities when the region is at its initiation stage or in small scale. Besides, due to the great contribution on self-reinforcement, lowering the risk compensation value for individuals is very important. The policy point can focus on improving entrepreneurial education, enhancing entrepreneurial experience so as to improve the individuals’ risk-taking abilities and creating fair businesses environment, supporting more resources to the individuals, or nurturing more business opportunities to downsize the risks in the regions.*

*5.3 Limitations and Direction for Future Research*

*One limitation of our study is confined to theoretical analysis, because robustness, validation and accuracy of our conclusions are not verified empirically. Our study adopts the model proceeded by mathematical analysis and numerical simulation, which can clearly exhibit the formation process of entrepreneurial agglomeration and conclude the determinants under strict model construction conditions, but have some weakness in introducing the real data to verify whether our conclusions can sufficiently reflect the real situations. Another limitation is that the determinants from our study are only confined to 4 categories; therefore, some other variables from prior research cannot be verified in our model. Our model has strong theoretical foundation, and most adopted determinants are suggested or mentioned by prior study, thus some of our findings also confirm to prior studies. However, limited by the mathematical model, we only adopt some of the variables; whether other variables could be regarded as the dynamics has not been proofed in our analysis.*

*Future research should modify the mathematical model, making it more acceptable to the variables suggested by prior research and observed from real situations, and more capable in explaining the mechanism of entrepreneurial agglomeration formation. In addition, empirical analysis combining with our theoretical model and using real data from various regions is also recommended in the future study.*

**2) Theoretical and practical implications is missing. Provide a more critical discussion regarding theoretical and practical implications. What are the theoretical and practical implications that can be derived from this study?
• Add practical implications
• Theoretical Implications**

**Response:** Thank you for the comment. We have refined the theoretical and practical implications and added them to our manuscript, all of which are marked in red color (under Section 5, page 27).

*5.2 Implications*

*Our findings have some theoretical implications: it is proved that entrepreneurship has “memory” and the entrepreneurial history could have influence on the future, which can be expressed as another kind of bandwagon effect emerging from entrepreneurial activities; it is concluded theoretically that 4 determinants from agents and regions, i.e., the agent’s risk compensation value, the initial scale of agents, the number of role models (especially in the early stage), and the initial entrepreneurial ratio, can contribute to the formation of entrepreneurial agglomeration, thus focusing on improving individual’s risk-taking abilities, increasing the initial population scale and entrepreneurial ratio in the region, and introducing more entrepreneur role models in the early age can improve the formation of entrepreneurial agglomeration.*

*Also, our findings have some practical implications. Considering the strong effects from “memory” and “history” in entrepreneurship, our policy should be more concentrated on the determinants from “early” age, such as attracting more role models and introducing more entrepreneurial activities when the region is at its initiation stage or in small scale. Besides, due to the great contribution on self-reinforcement, lowering the risk compensation value for individuals is very important. The policy point can focus on improving entrepreneurial education, enhancing entrepreneurial experience so as to improve the individuals’ risk-taking abilities and creating fair businesses environment, supporting more resources to the individuals, or nurturing more business opportunities to downsize the risks in the regions.*

At the end, we must thank you again. We hope this version of our paper would satisfy you.
