# Supplementary material for: The dynamics of entrepreneurial agglomeration formation: Social selection and simulation
Source: PLoS One. 2023 Sep 28;18(9):e0291615. doi: 10.1371/journal.pone.0291615 (PMC10538723; doi:10.1371/journal.pone.0291615)
Supplement: Attachment — Submitted filename: Response to REVIEWER.docx [file pone.0291615.s004.docx]

**Reviewer #1: Provide a more critical discussion regarding theoretical and practical implications. What are the theoretical and practical implications that can be derived from this study?**

**• Add practical implications**

**• Theoretical Implications**

**Response:** Dear reviewer, first of all, we would like thank the reviewer for giving valuable suggestions throughout the peer review process. Your comments really helped us to further improve the quality of the paper. We have refined the theoretical and practical implications and added them to our manuscript, all of which are marked in red color.

*“5.2 Implications*

*Practical implications*

*Firstly, considering the strong effects from “memory” and “history” in entrepreneurship, the policy should be more concentrated on the determinants from “early” age, such as attracting more role models and introducing more entrepreneurial activities when the region is at its initiation stage or in small scale. In detail, the government can start with the evaluation of a region’s entrepreneurial activities and make up polices to gather more role entrepreneurs and potential entrepreneurs into the region by cutting off taxes, providing more subsidies, or issuing more favorable measures to start and operate the businesses.*

*Secondly, due to the great contribution on self-reinforcement effect, lowering the risk compensation value for individuals is very important. The policy point can focus on fostering the traits on entrepreneurship. In detail, the government should invest more on improving entrepreneurship education, encouraging more potential entrepreneurs to observe, witness or participate in the incumbent business to enhance their entrepreneurial experience, so as to improve the individuals’ risk-taking abilities*

*Finally, for the reason that the improvement of entrepreneurial infrastructure is a good means to lower the entrepreneurship risks, and accordingly reduce the risk compensation value for the potential entrepreneurs. The policy can exert its ability on creating a fairer businesses environment, building more entrepreneurial facilities such as incubators, transportation and communication systems etc., to nurture more business opportunities and facilitate more entrepreneurial activities.*

*Theoretical implications*

*Firstly, we explore the dynamics of entrepreneurial formation. It is proved that the formation of entrepreneurial agglomeration is a self-reinforcing process. Based on social selection by individuals, we confirm that the formation of entrepreneurial agglomeration can be described as a Polya process, and the existing entrepreneurship will absorb or expel the new entrant. In our theoretical framework, the entrepreneurship has “memory” and the entrepreneurial history could have influence on the future, which can be expressed as another kind of bandwagon effect emerging from entrepreneurial activities.*

*Secondly, we explore the determinants which contribute to the entrepreneurial formation. Based on mathematical analysis and simulation, we conclude that some determinants from social selection can affect the formation of entrepreneurial agglomeration. In detail, our results theoretically indicate that 4 determinants from agents and regions, i.e., the agent’s risk compensation value, the initial scale of agents, the number of role models (especially in the early stage), and the initial entrepreneurial ratio, can contribute to the formation of entrepreneurial agglomeration, thus focusing on improving individual’s risk-taking abilities, increasing the initial population scale and entrepreneurial ratio in the region, and introducing more entrepreneur role models in the early age can improve the formation of entrepreneurial agglomeration.”*

Thank you for your comments!
